# Enhanced nitrogen removal via *Yarrowia lipolytica*-mediated nitrogen and related metabolism of *Chlorella pyrenoidosa* from wastewater

**DOI:** 10.3389/fbioe.2023.1159297

**Published:** 2023-06-22

**Authors:** Yuming Zhong, Danni Lin, Sufen Li, Qin Wang, Hui Liu, Lukai Ma, Huifan Liu

**Affiliations:** ^1^ College of Resources and Environment, Zhongkai University of Agriculture and Engineering, Guangzhou, Guangdong, China; ^2^ College of Light Industry and Food Technology, Zhongkai University of Agriculture and Engineering, Guangzhou, Guangdong, China; ^3^ Institute of Water Environment Engineering, Xinhua College of Sun Yat-Sen University, Guangzhou, Guangdong, China

**Keywords:** *Chlorella Pyrenoidosa*, co-cluture, *Yarrowia Lipolytica*, transcriptomics, nitrogen metabolism pathway

## Abstract

We investigated the optimum co-culture ratio with the highest biological nitrogen removal rate, revealing that chemical oxygen demand, total nitrogen (TN), and ammoniacal nitrogen (NH_3_-N) removal was increased in the *Chlorella pyrenoidosa* and *Yarrowia lipolytica* co-culture system at a 3:1 ratio. Compared with the control, TN and NH_3_-N content in the co-incubated system was decreased within 2–6 days. We investigated mRNA/microRNA (miRNA) expression in the *C. pyrenoidosa* and *Y. lipolytica* co-culture after 3 and 5 days, identifying 9885 and 3976 differentially expressed genes (DEGs), respectively. Sixty-five DEGs were associated with *Y. lipolytica* nitrogen, amino acid, photosynthetic, and carbon metabolism after 3 days. Eleven differentially expressed miRNAs were discovered after 3 days, of which two were differentially expressed and their target mRNA expressions negatively correlated with each other. One of these miRNAs regulates gene expression of cysteine dioxygenase, hypothetical protein, and histone-lysine N-methyltransferase SETD1, thereby reducing amino acid metabolic capacity; the other miRNA may promote upregulation of genes encoding the ATP-binding cassette, subfamily C (CFTR/MRP), member 10 (ABCC10), thereby promoting nitrogen and carbon transport in *C. pyrenoidosa*. These miRNAs may further contribute to the activation of target mRNAs. miRNA/mRNA expression profiles confirmed the synergistic effects of a co-culture system on pollutant disposal.

## 1 Introduction

The nitrogen and phosphorus accumulation in agriculture and industrial wastewater leads to water eutrophication (Judd et al., 2015). It is more challenging to treat this kind of wastewater using traditional biological treatment methods. The wastewater treatment using algae has become a promising approach with a high effect on biological nitrogen removal (BNR) (Vyverman, 2016). Many types of filamentous algae have been used in wastewater treatment, including *Oedogonium*, *Cladophora*, *Chlorella*, *Spirogyra*, and *Rhizoclonium* (Liu et al., 2020). Nevertheless, treating wastewater using algae alone is more expensive and inefficient. Co-culture of microalgae and microorganisms is a cost-effective and efficient technology (Li et al., 2022). Microalgae are photosynthetic microorganisms (Patidar et al., 2015) that can recover and recycle nutrients from wastewater and accumulate large amounts of valuable biomass (Nwoba et al., 2019). It absorbs nitrogen from wastewater through photosynthesis and releases oxygen for microorganisms. Microorganisms can use the organic pollutants in the wastewater and release carbon dioxide through respiration to enhance the photosynthesis of microalgae (Lee et al., 2015; Singh et al., 2020). BNR is reached while forming a benign energy-materials cycle. *Chlorella pyrenoidosa* is one of the strains with a high BNR efficiency, which can be co-cultured with various microorganisms to provide resource recovery (Liu et al., 2019), nitrogen deep treatment, and generation of novel bioactive substances associated with sewage systems (Vaca-Garcia, 2014). In our previous study, we confirmed that the intracellular protein has structural differences in the co-culture system of *C.pyrenoidosa* and *Yarrowia lipolytica* (3:1), showing potent antioxidant activity (QinWang et al., 2019). Furthermore, trypsin-hydrolyzed peptides were obtained in our study through this specific co-culture system, which exerts remarkable antioxidant activity (Liu H, 2019).

The microalgae-yeast symbiosis system highlights the significance of wastewater treatment. It was reported that the algae-assisted sequencing batch biofilm reactor increased the TN removal efficiency from 38.5% to 65.8% (385–658 mg/L) (He, 2018). The mixture of algae, ammonia-rich oxidizing bacteria, and methanol can rapidly remove nitrogen in the reactor. (He, 2018). Recently, researchers have been paying more attention to the algae-yeast co-culture for the economical improvement of microalgae biomass. For example, *Chlorella vulgaris (C. vulgaris)* and *Yarrowia lipolytica (Y. lipolytica)* co-culture has a strong potential for the treatment of industrial wastewater (Dong, 2018; Asraful, 2019). It was reported that the co-culture of *C.pyrenoidosa* and yeast improves the nitrogen removal rate (Huankai Li, 2019). Our previously established novel method using a co-culture of *C.pyrenoidosa* and *Rhodotorula glutinis* showed removal efficiencies of 58.53 and 36.07 for ammoniacal nitrogen (NH_3_-N) and total nitrogen (TN) in piggery wastewater. This is due to the fact that inorganic nitrogen N) is a necessary nutrient for microalgae, as it is required for the synthesis of proteins, polysaccharides, and other biomolecules and usually exists in the form of nitrate, nitrite, and ammonium (Stefan Schmollinger et al., 2014). Ammonium is the preferred form of nitrogen. In general, algae prefer ammonium and consume nitrate only when ammonium is almost depleted (Cai et al., 2013a). Yeast converts organic nitrogen compounds into N, and microalgae preferentially take up NH_3_-N, which is the main component of TN (about 60%) and the most important pollutant in wastewater (Vlaeminck et al., 2009), and convert it to substances for growth through nitrogen metabolism. We consider that microalgae and yeast can exhibit a mutually beneficial relationship in the co-culture system and the co-culture system would affect microalgae nitrogen metabolism and gene expression, so as to enhance nitrogen removal in wastewater. MicroRNAs (miRNAs) are key regulators in gene expression that can control gene expression via messenger RNA (mRNA) cleavage or translational repression (Djami-Tchatchou et al., 2017; Lou et al., 2018). Meanwhile, miRNA-target genes are primarily involved in secondary metabolite biosynthesis (Azaman et al., 2020). Microalgae nitrogen metabolism can be influenced by both miRNA and mRNA, and the presence of yeast stimulates gene expression in microalgae. The mechanism of the interaction between the two species, however, is currently unclear.

Therefore, this study aimed the following: 1) Establishment of a co-culture system of *C. pyrenoidosa* and *Y. lipolytica* at different ratios (1:0, 1.5:1, 3:1, 6:1) for 6 days in stimulated wastewater, to find out the optimum ratio of the co-culture system with the highest BNR; 2) daily assessment of the BNR for the optimum co-culture ratio in stimulated wastewater to confirm the best day with the highest BNR; 3) identification of significant transcriptomic differences in metabolic pathways and systematical analysis of the significant differences in transcription pathways regulated by miRNAs, including nitrogen, amino acids, photosynthetic, and carbon metabolism; 4) quantitative PCR analysis of key genes for verification. This study provides some useful information on efficient BNR under the algal-organism symbiosis system.

## 2 Materials and methods

### 2.1 Microalgae and cultivation conditions

**FIGURE 1:**
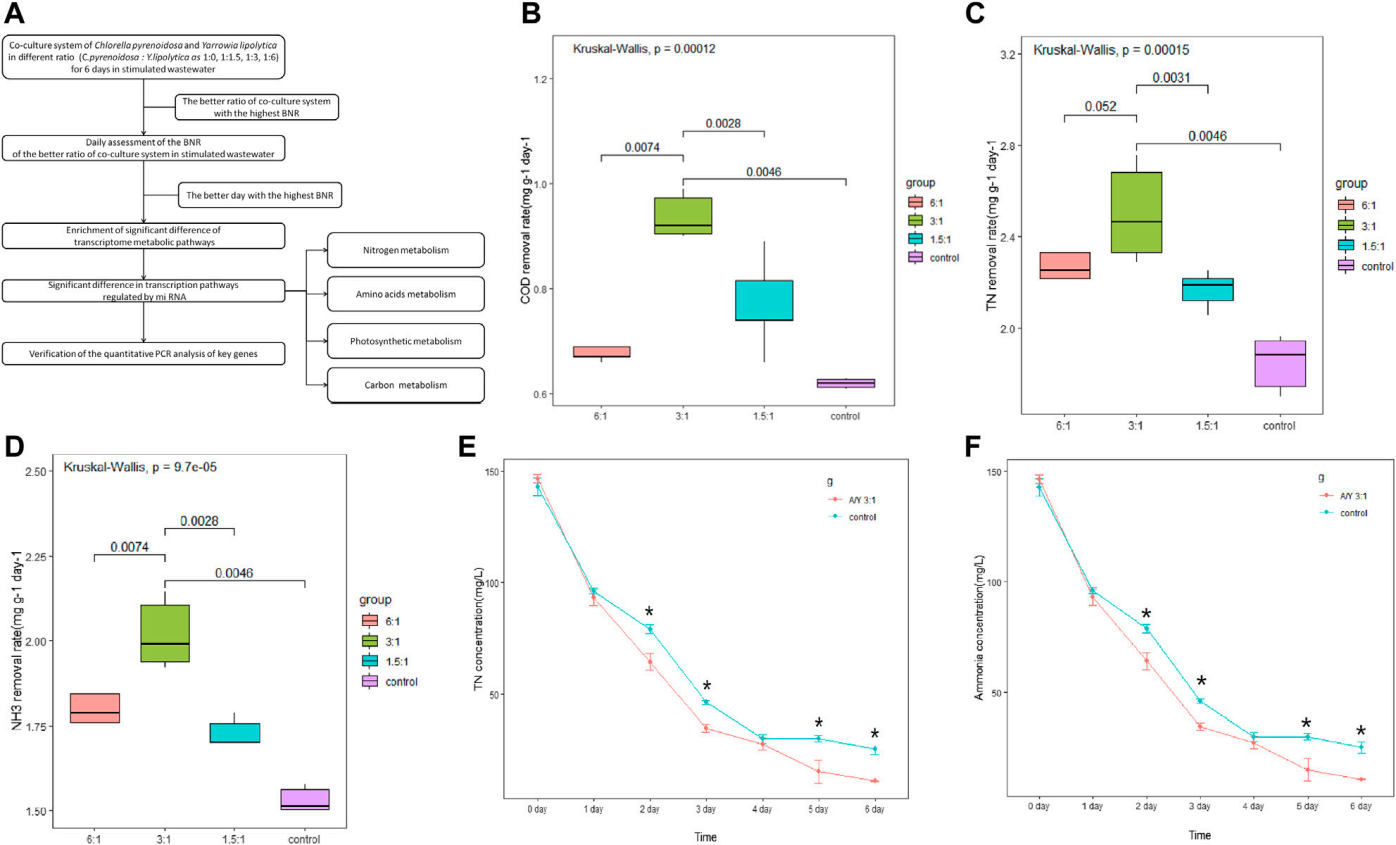
Cocultures of *Y. lipolytica* with Chlorella species. **(A)** The summarized experimental process, **(B)** COD removal rate, **(C)** TN removal rate, **(D)** NH_3_-N removal rate, **(E)** concentration of TN and **(F)** change in the amount of ammonia during 6 days of co-culture tests. Error bars represent the standard deviation of three cultures and the Kruskal test was performed.

Figure 1A shows the summarized experimental process. *Chlorella pyrenoidosa* was obtained from the Institute of Hydrobiology, Chinese Academy of Sciences, cultured in sterile Tris-acetate phosphate (TAP) medium. *Y. lipolytica* was obtained from the Laboratory of Guangdong Institute of Microbiology) and cultured in yeast mold medium (YM; Qingdao Hope Bio-Technology Co., Ltd.). They were placed in a light incubator and cultured in the light environment at 25°C, 36 μE/m^2^/s, shaking at 150 rpm.

The activated *C. pyrenoidosa* and *Y. lipolytica* were obtained after 3 and 2 days of incubation, respectively. The active *C. pyrenoidosa* and the active *Y. lipolytica* were mixed in 200 mL sterile stimulation wastewater at the ratio of 1: 0, 1.5: 1, 3: 1, and 6: 1 respectively for 6 days. The density of algae cells is maintained at 0.10–10.00×10^5^ cells/mL (Cadena-Herrera et al., 2015). Refer to the article of Huankai Li (Li et al., 2019) to configure the nutrient concentration in the simulated wastewater. The content of chemical oxygen demand (COD), TN, and NH_3_-N were determined every day.

The growth curves were consistent with our previous studies, and the biomass yield of *C. pyrenoidosa* and *Y. lipolytica* peaked at an inoculation ratio of 3:1 (Huankai Li, 2019; Wang et al., 2019). After 3 and 5 days, active *C. pyrenoidosa* and active *Y. lipolytica* were mixed in sterile 200 mL Tris-acetate phosphate (TAP) medium (Wang et al., 2019) at the ratio of 1:0 and 3:1, respectively. The initial inoculation density was 5.00×10^5^ cells/mL. The algal biomass was collected and transcriptome analysis was performed.

### 2.2 Parameters measurement

The concentration of the nutrients and other parameters of stimulated wastewater were measured, including COD, TN, and NH_3_-N (Liu et al., 2016). Where TN was measured on a TN analyzer (Jane Multi N/C 3000, Germany), COD and NH_3_-N were measured according to APHA standard methods (Gilcreas, 1966).

### 2.3 mRNA extraction and sequencing

*C. pyrenoidesa* cells and the co-culture system on days 3 and 5 were collected via centrifugation. Total RNA was obtained from *C. pyrenoidesa* and the co-culture system using the TRIzol reagent (Invitrogen, Carlsbad, CA, United States) following the manufacturer’s procedure, and sequencing libraries were generated using the NEBNext®Ultra™ RNA Library Prep Kit for Illumina^®^ (NEB, United States). Briefly, the process includes the repair of fragmented DNA ends, the addition of A-tailing to the 3′ends, ligation adaptor, and PCR amplification. cDNA fragments were amplified using the AMPure XP system (Beckman Coulter, Beverly, United States). Finally, the Illumina HiSeq4000 (LC Sciences, United States) high throughput sequencing platform was used for sequencing.

### 2.4 mRNA expression analysis and functional annotation

Clean data in fastq format were obtained by removing reads containing the adapter, the ploy-N, and low-quality reads from the raw data through in-house Perl scripts. The reads were assembled using the Trinity software (Anders and Huber, 2010). The Unigene library was compared using bowtie software, and the transcriptional abundance was analyzed using RSEM software (Li and Dewey, 2011). Unigene expression was calculated and normalized to fragments per kilobase of transcript per million mapped reads (FPKM). The resulting *p*-values were adjusted using Benjamini and Hochberg’s approach for controlling the false discovery rate (FDR) (Elowitz et al., 2002; Rajkumar et al., 2015). FDR<0.01 and fold change ≥2 were taken as filtering criteria for the differentially expressed genes (DEGs). The DEGs were compared using Nr, SwissProt, GO, KOG, EggNOG, and KEGG databases with blast software to obtain gene annotation information (Elowitz et al., 2002).

### 2.5 miRNA library construction

Total RNA was extracted from *C. pyrenoidesa* and the co-culture system using the TRIzol reagent (Invitrogen, Carlsbad, CA, United States) with the manufacturer’s protocol, and the miRNA sequencing library was generated using the Next Ultra-small RNA Sample Library Prep Kit for Illumina (NEB, United States). The library quality was determined using Agilent 2100 Bioanalyzer (Beckman Coulter, Beverly, United States) (Masotti and Preckel, 2006). Finally, the library was sequenced using the Illumina hiseq (LC Sciences, United States) high throughput sequencing platform.

### 2.6 miRNA expression analysis

Raw data in the fastq format were processed (Zhao et al., 2019) and clean data were obtained using the method described above for mRNAs. Clean data were obtained by removing the sequences smaller than 18 nucleotides (nt) or longer than 30 nt. The Q20, Q30, and GC content were evaluated the quality of sequencing. The mapped small RNA tags were then compared using the Bowtie tools (Langmead et al., 2009), and clean reads were aligned using the Silva, GtRNAdb, Rfam, and Repbase databases to remove certain RNA categories including rRNA, transfer RNA (tRNA), small nuclear RNA (snRNA), small nucleolar RNA (snoRNA), and other non-coding RNAs, as well as repeats. The known miRNA and novel miRNAs were obtained using the known miRNA database and mirdeep2 software, respectively. Then, the expression of miRNA was measured by the number of transcripts per million clean tags (TPM) (Mortazavi et al., 2008). |log2(FC)|≥0.58 was set as the threshold for a significantly differential expression. The target genes corresponding to miRNAs were predicted using Target Finder software.

### 2.7 Quantitative real-time PCR (qRT-PCR) analysis

The expression of several typical mRNAs and miRNAs was measured using qRT-PCR to verify the reliability of transcriptome sequencing data (Jozefczuk and Adjaye, 2011). The primers were designed using the Primer Premier 5.0 software (Ren et al., 2004), and mRNA and miRNA information for qRT-PCR are shown in Supplementary Table S1. The relative transcript abundance was calculated using the 2^−ΔΔCT^ method (Jianhua et al., 2016). Each sample was analyzed in triplicates.

### 2.8 Statistical analysis

All experiments were conducted in triplicates. The mean values along with standard deviations were calculated. The significant difference was determined using Kruskal tests. Stars indicate significant differences between treatments (*p* < 0.05).

Heatmap, Volcano Plot, and bar plots were established using R packages pheatmap and ggplot2. The correlation tests were performed and verified using R packages psych and vegan. Other plots and analyses were conducted using R 3.52 and R packages.

## 3 Results and discussion

### 3.1 BNR-associated removal by the C*.pyrenoidosa and Y.lipolytica* co-culture system

The growth rate of *Y.lipolytica* in the symbiotic system is faster than that of *C. pyrenoidosa*, and it holds a dominant position in 6 days (Wang et al., 2019). The biomass yield reached the peak when the inoculation ratio of *Y.lipolytica* to *C. pyrenoidosa* was 3: 1, and reached 5.35 g/L after 6 days (Huankai Li, 2019). To analyze the effect of *Y.lipolytica* on the degradation of different organic compounds in single and mixed culture systems, we measured the COD, TN, and NH_3_-N removal rates on day 6 of incubation at 1.5:1, 3:1, and 6:1 ratios and the results are shown in Figures 1B–F. Results suggested that the COD removal rate was significantly increased in the co-incubation system, especially with the co-culture of *C. pyrenoidosa* to *Y. lipolytica* at a 3:1 ratio (Figure 1B), and the removal rate reached 56.25% after 6 days of culture, from 1,200 mg/L to 525 mg/L. Some studies have shown that 50%–90% (500–900 mg/L) COD can be removed by *C. pyrenoi*dosa (Gupta et al., 2017; Li et al., 2019; Bai et al., 2022). However, it seems that COD cannot be completely removed only by *C. pyrenoidosa*, which is probably due to the COD content of the refractory organic compounds as a result of the anaerobic respiration in the *C. pyrenoidosa* culture (Xia and Murphy, 2016). Compared with a single culture system, the co-culture system had significant advantages in wastewater pollutant removal, which may be because *Y. lipolytica* degraded these compounds so that they can be utilized by *C. pyrenoidosa*. As for nitrogen, the co-incubated system also increased the TN and NH_3_-N removal rate, especially at a 3:1 ratio (Figures 1C,D). Compared with the control, the TN and NH_3_-N content in the co-incubation system at a 3:1 ratio was significantly decreased within 2–6 days (Figures 1E, F). The removal of TN and NH_3_-N from the wastewater after 6 days of co-cultivation was 147.82 mg/L and 98.54 mg/L, respectively. Both removal efficiencies were above 90.00%, and the changing trend of NH_3_-N was similar to that of TN. Nitrogen is an essential element for the synthesis of various biological substances, such as proteins, nucleic acids, and phospholipids (Xu et al., 2015). NH_3_-N is the major component of TN in the wastewater and it is a major source of nitrogen in wastewater, which was considered the utilizable form of nitrogen for *C. pyrenoidosa* and *Y. lipolytica* growth (Cai et al., 2013a). Some studies have shown that the difference in ammonia stripping and assimilative capacity may be the reason why the removal efficiency of the co-culture system was higher than that of the single culture (Qin et al., 2018).

### 3.2 Differential expression of the transcriptome in response to *Y.lipolytica*

RNA-seq of six libraries from *C. pyrenoidosa* cultured for 5 days with and without *Y. lipolytica* resulted in 186.48 Gb of clean data, with >93.82% of the data exhibiting a quality score of Q30 (Supplementary Table S2). Using Trinity, 258,147 high-quality clean reads were assembled *de novo* into 34,010 unigenes with a mean length of 934.53 base pairs (bp) and an N50 length of 1704 bp. The length of all unigenes was >200 bp and the length of 29.53% (10,044) of the unigenes was >1,000 bp (Supplementary Table S3), which indicated that the obtained transcriptome data were acceptable and reliable with good quality.

**FIGURE 2:**
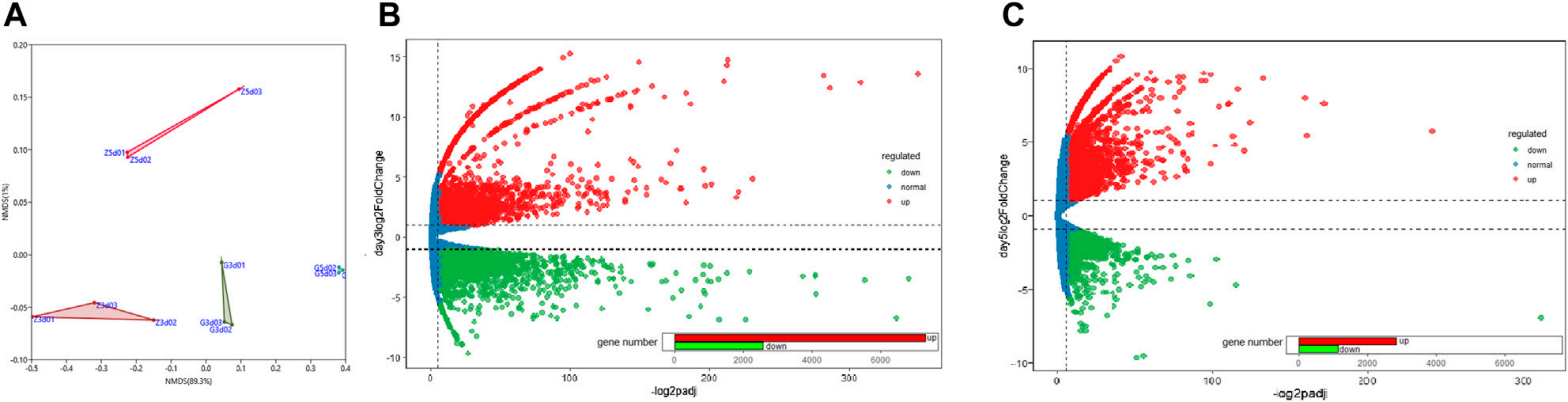
**(A)** NMDS plots of differentially expressed genes. MA plots of differentially expressed genes after **(B)** 3 and **(C)** 5 days of culture.

The expression levels of 12 libraries from *C.pyrenoidosa* with or without *Y.lipolytica* after 3 or 5 days, which were calculated based on the FPKM values, showed that the incubation time and *Y. lipolytica* had a certain impact on the transcriptome of *C. pyrenoidosa* (Figure 2A). Based on the expression levels, we identified DEGs. There were 9885 DEGs, including 2575 downregulated and 7310 upregulated genes for *C. pyrenoidosa* after 3 days (Figure 2B). Exactly 3976 DEGs were detected, including 1,149 downregulated and 2827 upregulated genes for *C. pyrenoidosa* after 5 days (Figure 2C). The functional annotation of *C. pyrenoidosa* unigenes with or without *Y. lipolytica* co-culture in each database is listed in Supplementary Table S4. Therefore, *Y.lipolytica* had a great effect on gene expression in *C.pyrenoidosa*, especially after 3 days of culture.

### 3.3 The potential nitrogen metabolism of the co-culture system

**FIGURE 3:**
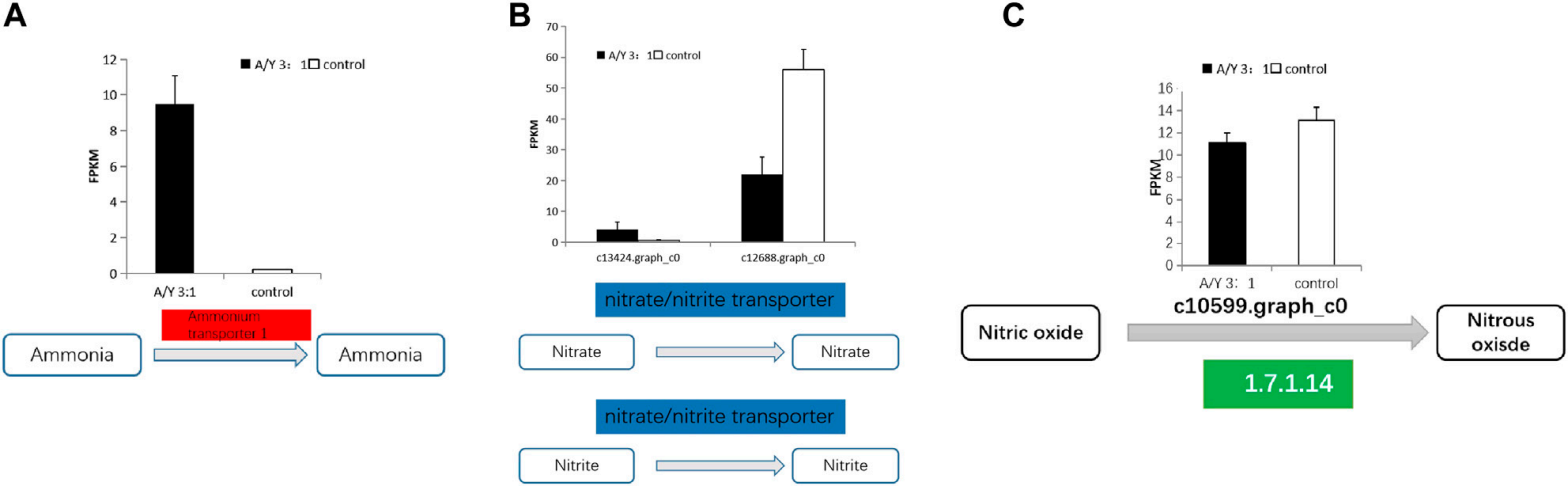
The representative differentially expressed genes associated with **(A–C)** the nitrogen metabolism of *C. pyrenoidesa* in response to *Y. lipolytica*.

Carbon and nitrogen metabolisms were identified as the center metabolic pathways, as well as photosynthesis, in *C. pyrenoidosa*. Exactly 4 DEGs were involved in the nitrogen metabolism, which are displayed in Figure 3 and Table 1, such as genes encoding ammonia transporter, nitrate/nitrite transporter, and fungal nitric oxide reductase. According to the biomass analysis, *Y. lipolytica* could significantly improve TN and ammonia removal rates (Figures 1C–F). Compared with a single culture system, ammonia transporter-related genes were upregulated, nitrate/nitrite transporter-related genes were mix-regulated, and fungal nitric oxide reductase was downregulated in the co-culture system (Figure 3).

**TABLE 1:** Genes involved in nitrogen metabolism from *C. pyrenoidosa* in co-culture system.

#ID	KEGG_annotation	KEGG_pathway	EC number	FDR	Log2FC	regulated
c13453.graph_c3	K03320 ammonium transporter, Amt family	Ammonium transporter 1 member 1		1.96E-51	4.53793	up
c13424.graph_c0	K02575 MFS transporter, NNP family, nitrate/nitrite transporter	Nitrogen metabolism (ko00910)	-	1.88E-08	2.353473	up
c12688.graph_c0	K02575 MFS transporter, NNP family, nitrate/nitrite transporter	Nitrogen metabolism (ko00910)	-	2.35E-10	−2.18646	Down
(unconservative_c10657.graph_c1_1707)						
c10599.graph_c0	K15877 fungal nitric oxide reductase [EC:1.7.1.14] | (RefSeq) CYP55B1; cytochrome P450, nitric oxide reductase	Nitrogen metabolism (ko00910)	1.7.1.14	3.23E-10	−1.15526	down

Ammonia and nitrate transport were identified as the main pathway of nitrogen assimilation and nitrogen metabolism in *C. pyrenoidosa*. Because ammonia was the only nitrogen source in our batch tests, the ammonia transporter was related to varying nitrogen sources and levels (Klahn et al., 2015). Nitrate/nitrite is transported into the cell by a nitrate transporter (Mtolera, 2003; Sanz-Luque et al., 2013). Nitrate reductase activity was related to the content of nitrate, so it can control the nitrate assimilation rates (Scherholz and Curtis, 2013). Nitric oxide (NO) is reduced to nitrous oxide (N2O) by fungal nitric oxide reductase in the NO pathway (Zhang and Shoun, 2008). The ammonia transporter and nitrate transporter were upregulated by *Y. lipolytica*. We inferred that *Y. lipolytica* fixed nitrogen and might provide nitrogen for *C. pyrenoidosa*, leading to the change of nitrogen form and concentration in the co-culture system in the co-culture system. A similar observation has been reported between *Chlorella variabilis* and *Rhizobium* (CongFei et al., 2019).

### 3.4 Potential photosystem and carbon metabolism of the co-culture system

**FIGURE 4:**
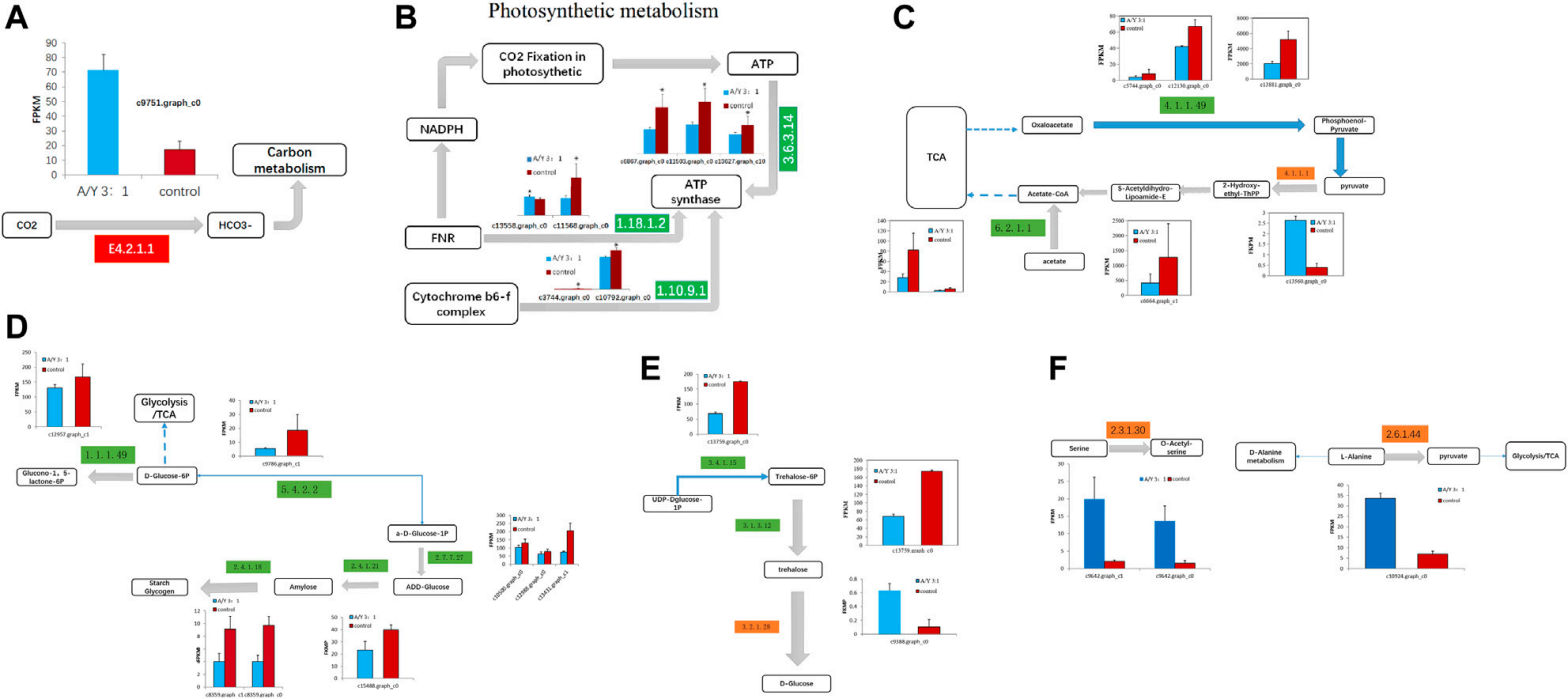
The representative differentially expressed genes associated with **(A–B)** the photosynthesis metabolism, **(C)** TCA cycle, **(D–E)** glycometabolism, and **(F)** amino acid metabolism of *C. pyrenoidesa* in response to *Y. lipolytica*.

After ammonia was transported into the cell, we also identified 1 DEG related to carbon metabolism and 11 DEGs related to photosystem metabolism as shown in Figures 4A, B and Table 2. Photosystem metabolism was mainly related to F-type H + -transporting ATPase subunit b, photosystem II oxygen-evolving enhancer protein 1, cytochrome b6-f complex iron-sulfur subunit, ferredoxin--NADP + reductase, F-type H + -transporting ATPase subunit gamma, ferredoxin, cytochrome b6-f complex subunit 8, and carbonic anhydrase activity. Assimilation carbon pathways were identified as other associate pathways related to the nitrogen pathway. Combined with the biomass analysis, *Y. lipolytica* could significantly increase the removal rate of COD (Figure 1C). *Y. lipolytica* could significantly enhance the expression level of carbonic anhydrase (EC:4.2.1.1), indicating that the conversion of carbon dioxide to HCO^3−^ strengthened under the effect of CO_2_ assimilation in the co-culture system (Figure 4A). It has been reported that *C. pyrenoidosa* and other microalgae can utilize CO_2_ by yeast respiration (Lei et al., 2018). Yeast oxidizes and decomposes carbonaceous organic matter to CO_2_. Some of the CO_2_ escapes into the atmosphere, while some is absorbed and utilized by microalgae cells. O_2_ generated by microalgae photosynthesis enhances yeast’s oxidative decomposition of carbonaceous organic matter, allowing inorganic carbon to be removed from wastewater. As a result of algal photosynthesis consuming a large amount of acidic CO_2_, the pH of the wastewater rises and becomes alkaline, and some N is removed by overflowing as NH_3_ (Vadiveloo et al., 2020). Besides, a series of genes of *C. pyrenoidosa* were downregulated in the photosynthesis pathway (Figure 4B), which could limit the synthesis of NADPH and ATP required for dark reactions (Markou et al., 2016). Therefore, we inferred that the presence of *Y. lipolytica* in the mixed culture reduced the available light for *C. pyrenoidosa*, limited its photosynthesis, and further limited the fixation of CO_2_ by *C. pyrenoidosa*. *Y. lipolytica* enhanced the exchange between oxygen and CO_2_ (Zhang et al., 2018a). Therefore, CO_2_ and organic matter from *Y. lipolytica* and COD in wastewater may be used as a carbon source for *C. pyrenoidosa* growth.

**TABLE 2:** Genes involved in Photosynthetic metabolism by co-culture system of *C.pyrenoidosa* and *Y.lipolytica*.

#ID	KEGG_annotation	KEGG_pathway	EC number	FDR	log2FC	regulated
c9751.graph_c0	K01672 carbonic anhydrase	Nitrogen metabolism (ko00910)	4.2.1.1	8.18E-08	1.298964	up
c6867.graph_c0	K02109 F-type H + -transporting ATPase subunit b	Oxidative phosphorylation (ko00190); Photosynthesis (ko00195)	3.6.3.14	1.31E-06	−1.88311	down
c11359.graph_c0	K02716 photosystem II oxygen-evolving enhancer protein 1	Photosynthesis (ko00195)	1.10.9.1	0.000374	−1.15225	down
c10792.graph_c0	K02636 cytochrome b6-f complex iron-sulfur subunit	Photosynthesis (ko00195)	1.18.1.2	0.000269	−1.05979	down
c11568.graph_c0	K02641 ferredoxin--NADP + reductase	Photosynthesis (ko00195)	3.6.3.14	4.11E-06	−2.09084	down
c11503.graph_c0	K02115 F-type H + -transporting ATPase subunit gamma	Oxidative phosphorylation (ko00190); Photosynthesis (ko00195)	1.18.1.2	2.67E-06	−1.78285	down
c13558.graph_c0	K02641 ferredoxin--NADP + reductase	Photosynthesis (ko00195)	3.6.3.14	0.000196	−1.00568	down
c13627.graph_c10	K02113 F-type H + -transporting ATPase subunit delta	Oxidative phosphorylation (ko00190); Photosynthesis (ko00195)	-	0.000169	−1.5084	down
c13836.graph_c5	K02639 ferredoxin	Photosynthesis (ko00195)	-	8.02E-06	−1.34609	down
c10636.graph_c0	K02639 ferredoxin	Photosynthesis (ko00195)	-	4.25E-08	−4.04499	down
c4457.graph_c0	K03689 cytochrome b6-f complex subunit 8	Photosynthesis (ko00195)	-	7.18E-08	−1.57106	down
c10483.graph_c0	K02639 ferredoxin	Photosynthesis (ko00195)	-	1.45E-08	−2.53684	down

Glucose was a key material of the stress response, as well as carbon and nitrogen metabolisms, which was related to the phosphorylation of glucose by regulating gene expression in signal transduction pathways and a variety of plant hormone signal pathways (Moore and Rolland, 2003). Meanwhile, glycolysis/gluconeogenesis, pentose phosphate, starch, and sucrose pathway-related genes encoding two potentially rate-limiting enzymes, phosphoglucomutase (EC:5.4.2.2) and glucose-6-phosphate 1-dehydrogenase (EC: 1.1.1.49), were downregulated after *Y. lipolytica* treatment. Due to several downregulated genes, D-glucose-6P might not be converted to glucono-1, 5-lactone-6P, and D-glucose-1P, which is related to the starch/glycogen synthesis (Figure 4C; Table 3). D-glucose-6P is related to the tricarboxylic acid cycle (TCA) and synthesis. Moreover, glucose 6 phosphate dehydrogenase (EC: 1.1.1.49) was the first rate limiting enzyme in the well-known pentose phosphate oxidation pathway, catalyzing glucono-1,5-lactone-6P and gluconate synthesis (Huntzinger and Elisa, 2011). These reactions produce moieties of reducing equivalents, such as NADPH, which is utilized for nitrogen assimilation in algae (Zhang et al., 2018b). The upregulation of the glycolysis reactions of fructose and mannose and trehalose metabolism with the genes of carbon metabolism, may suggest an ammonia transport that increased the applicability of carbon and further promoted the synthesis of amino acids.

**TABLE 3:** Genes involved in TCA cycle by co-culture system of *C.pyrenoidosa* and *Y.lipolytica*.

#ID	KEGG annotation	KEGG pathway	EC number	FDR	log2FC	regulated
c5744.graph_c0	K01610 phosphoenolpyruvate carboxykinase (ATP)	Glycolysis/Gluconeogenesis (ko00010); Citrate cycle (TCA cycle) (ko00020); Pyruvate metabolism (ko00620); Carbon fixation in photosynthetic organisms (ko00710); Carbon metabolism (ko01200)	4.1.1.49	0.001694	−1.85458	down
c13881.graph_c0	K01610 phosphoenolpyruvate carboxykinase (ATP)	Glycolysis/Gluconeogenesis (ko00010); Citrate cycle (TCA cycle) (ko00020); Pyruvate metabolism (ko00620); Carbon fixation in photosynthetic organisms (ko00710); Carbon metabolism (ko01200)	4.1.1.49	3.85E-20	−2.25509	down
c12130.graph_c0	K01610 phosphoenolpyruvate carboxykinase (ATP)	Glycolysis/Gluconeogenesis (ko00010); Citrate cycle (TCA cycle) (ko00020); Pyruvate metabolism (ko00620); Carbon fixation in photosynthetic organisms (ko00710); Carbon metabolism (ko01200)	4.1.1.49	1.73E-08	−1.62631	down
c6664.graph_c1	K01895 acetyl-CoA synthetase	Glycolysis/Gluconeogenesis (ko00010); Pyruvate metabolism (ko00620); Propanoate metabolism (ko00640); Carbon metabolism (ko01200)	6.2.1.1	7.57E-08	−2.57417	down
c6664.graph_c0	K01895 acetyl-CoA synthetase	Glycolysis/Gluconeogenesis (ko00010); Pyruvate metabolism (ko00620); Propanoate metabolism (ko00640); Carbon metabolism (ko01200)	6.2.1.1	3.14E-07	−2.54898	down
c5014.graph_c0	K01895 acetyl-CoA synthetase	Glycolysis/Gluconeogenesis (ko00010); Pyruvate metabolism (ko00620); Propanoate metabolism (ko00640); Carbon metabolism (ko01200)	6.2.1.1	0.000108	−2.07398	down
c13560.graph_c0	K01568 pyruvate decarboxylase	Glycolysis/Gluconeogenesis (ko00010)	-	1.51E-07	1.877819	up

Meanwhile, the results of carbon metabolic analysis showed that the mixed culture with *Y. lipolytica* weakened the photosystem of *C. pyrenoidosa* and strengthened glycolysis/gluconeogenesis and starch pathways. The CO_2_ fixing pathway was downregulated and CO_2_ assimilation was enhanced in *C. pyrenoidosa*, which would deeply affect other carbon pathways, such as glycolysis/gluconeogenesis and starch synthesis. In starch synthesis, alpha and alpha-trehalase [EC:3.2.1.28] were upregulated after *Y. lipolytic*a treatment. Trehalose conversion to D-glucose was enhanced, which suggested that glucose came from the trehalose storage, while the trehalose synthesis pathway was weakened in the mixed culture (Figure 4D; Table 4).

**TABLE 4:** Genes involved in carbon metabolism by co-culture system of *C.pyrenoidosa* and *Y.lipolytica*.

#ID	KEGG_annotation	KEGG_pathway	EC number	FDR	log2FC	regulated
c12957.graph_c1	K00036 glucose-6-phosphate 1-dehydrogenase	Pentose phosphate pathway (ko00030); Glutathione metabolism (ko00480); Carbon metabolism (ko01200)	1.1.1.49/1.1.1.363	1.41E-26	−1.65064	down
c10500.graph_c0	K00975 glucose-1-phosphate adenylyltransferase	Starch and sucrose metabolism (ko00500); Amino sugar and nucleotide sugar metabolism (ko00520)	2.7.7.27	6.56E-13	−1.21775	down
c12988.graph_c0	K00975 glucose-1-phosphate adenylyltransferase	Starch and sucrose metabolism (ko00500); Amino sugar and nucleotide sugar metabolism (ko00520)	2.7.7.27	3.28E-11	−1.22411	down
c13431.graph_c1	K00975 glucose-1-phosphate adenylyltransferase	Starch and sucrose metabolism (ko00500); Amino sugar and nucleotide sugar metabolism (ko00520)	2.7.7.27	5.00E-33	−2.29692	down
c8359.graph_c1	K00700 1,4-alpha-glucan branching enzyme	Starch and sucrose metabolism (ko00500)	2.4.1.18	6.55E-08	−2.15997	down
c8359.graph_c0	K00700 1,4-alpha-glucan branching enzyme	Starch and sucrose metabolism (ko00500)	2.4.1.18	6.32E-12	−2.22363	down
c4935.graph_c0	K00703 starch synthase	Starch and sucrose metabolism (ko00500)	2.4.1.21	1.21E-07	−1.74405	down
c9388.graph_c0	K01194 alpha,alpha-trehalase	Starch and sucrose metabolism (ko00500)	3.2.1.28	0.00695	2.08054	up
c9786.graph_c1	K01835 phosphoglucomutase	Glycolysis/Gluconeogenesis (ko00010); Pentose phosphate pathway (ko00030); Galactose metabolism (ko00052); Purine metabolism (ko00230); Starch and sucrose metabolism (ko00500); Amino sugar and nucleotide sugar metabolism (ko00520)	5.4.2.2	6.12E-10	−2.5763	down

### 3.5 Potential amino acid biosynthesis metabolism of the co-culture

Amino acids are one of the organic nitrogen sources for microalgae (Näsholm et al., 2009), which contain a large number of amino acid transporters (Rentsch et al., 2007). Amino acids are degraded to provide ammonium, which is then taken up by microalgae and contributes to the denitrification of wastewater (Serna and Martin, 2006). Co-culture systems can promote amino acid synthesis in microalgae (Zhang et al., 2011). Exactly six DEGs were involved in amino acid biosynthesis, which are displayed in Figure 4F and Table 4, such as genes encoding serine O-acetyltransferase, alanine-glyoxylate transaminase/(R)-3-amino-2-methylpropionate-pyruvate transaminase, glutamine synthetase, and cysteine dioxygenase. In the co-culture, alanine-glyoxylate transaminase/(R)-3-amino-2-methylpropionate-pyruvate transaminase expression was upregulated 1.35 times, which is related to alanine aspartate and glutamate metabolism. It controls the conversion from L-alanine to pyruvate, which is related to the D-alanine metabolism and TCA cycle. Glutamine synthetase, a key enzyme in amino acid metabolism, is regulated in multiple ways in response to varying nitrogen sources and levels. Some studies have shown that the absorption of NH_3_-N is an important process of arginine synthesis in *C. pyrenoidosa* (Llacer et al., 2007). We found two kinds of glutamine synthetases in the co-culture, which were downregulated. The results showed that *Y. lipolytica* and *C.pyrenoidosa* could choose different ammonia sources for the glutamate metabolism. Both *Y. lipolytica* and *C.pyrenoidosa* could achieve assimilation of ammonia, which contributed to the synthesis of different amino acids.

### 3.6 miRNA regulation in the co-culture

**FIGURE 5:**
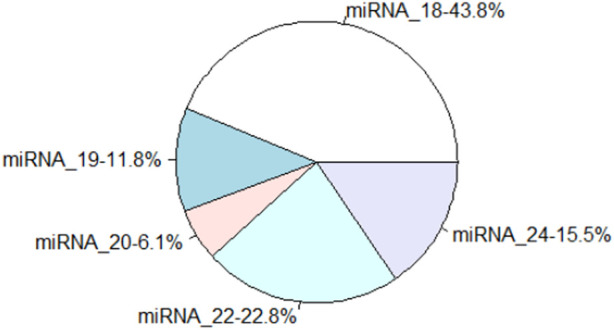
The length distributions of unique miRNA sequences.

Several miRNAs from *C.pyrenoidosa* in the control and co-culture were identified. The low quality and contaminated reads, adaptor sequences, poly-A-containing sequences, and sequences shorter than 14 nt were removed, and more than 10 million clean reads were obtained in each sample, with Q30 ≥ 85%. Moreover, the length distribution of miRNAs in this study was mainly n 18–24 nt, and 18, 22, and 24 nt miRNAs showed a higher abundance, which accounted for 43.8, 22.8, and 15.5%, respectively (Figure 5A). miRNAs mainly play a role in regulating gene expression in many eukaryotes (Goossens et al., 2003).

In the present study, we identified 11 differentially expressed *C. pyrenoidesa* miRNAs after 3 days, including three upregulated and eight downregulated miRNAs. According to the expression correlation between miRNAs and their targets, some of them had the same change in expression, which may involve other regulatory mechanisms (Huntzinger and Elisa, 2011). However, two miRNA/mRNA pairs had the opposite trend (Table 5), which mainly focused on amino acid biosynthesis and the ABC transport pathway (Zhang et al., 2018b). *Y. lipolytica* activated the expression of unconservative_c13405.graph_c0_13924, which plays a role in regulating the expressions of its predicted target mRNA, including cysteine dioxygenase, hypothetical protein, and histone-lysine N-methyltransferase SETD1. The catabolism of cysteine was catalyzed by cysteine dioxygenase (CDO), which was the first major step in cysteine catabolism (Chai et al., 2006). Histone-lysine N-methyltransferase SETD1 mediates lysine methylation or demethylation (Florian Maumus et al., 2011). Therefore, it may reduce the metabolic capacity of amino acids by regulating the expression of related genes via unconservative_c13405.graph_c0_13924. Furthermore, the unconservative_c11633.graph_c0_4666 may further contribute to the upregulation of genes encoding the ATP-binding cassette, subfamily C (CFTR/MRP), member 10 (ABCC10), which play important roles in ABC transport (Duan et al., 2019). Peptides, carbohydrates, lipids, heavy metal chelates, inorganic acids, steroids, and xenobiotics are transported by ABC transporter proteins (Alain et al., 2003). Therefore, *Y. lipolytica* may promote nitrogen and carbon transport in *C. pyrenoidesa*.

**TABLE 5:** Correlations analysis between miRNAs and mRNAs by co-culture system of *C.pyrenoidosa* and *Y.lipolytica*.

miRNA name	Target gene	miRNA log2 fold change	*p*-value	mRNA log2 fold change	*p*-value	Target annotation
unconservative_c13405.graph_c0_13924	c13678.graph_c4	−0.947	0.009	1.523	4.17E-05	K00456 cysteine dioxygenase [EC:1.13.11.20]
	c8987.graph_c0	−0.947	0.009	2.519	0.0004	hypothetical protein
	c8785.graph_c0	−0.947	0.009	2.462	5.83E-18	K11422 histone-lysine N-methyltransferase SETD1 [EC:2.1.1.43]
unconservative_c11633.graph_c0_4666	c10576.graph_c1	−5.44147	0.00956	2.124808	0.007	K05674 ATP-binding cassette, subfamily C (CFTR/MRP), member 10

### 3.7 Verification of miRNA and mRNA expression profiles via qRT-PCR

**FIGURE 6:**
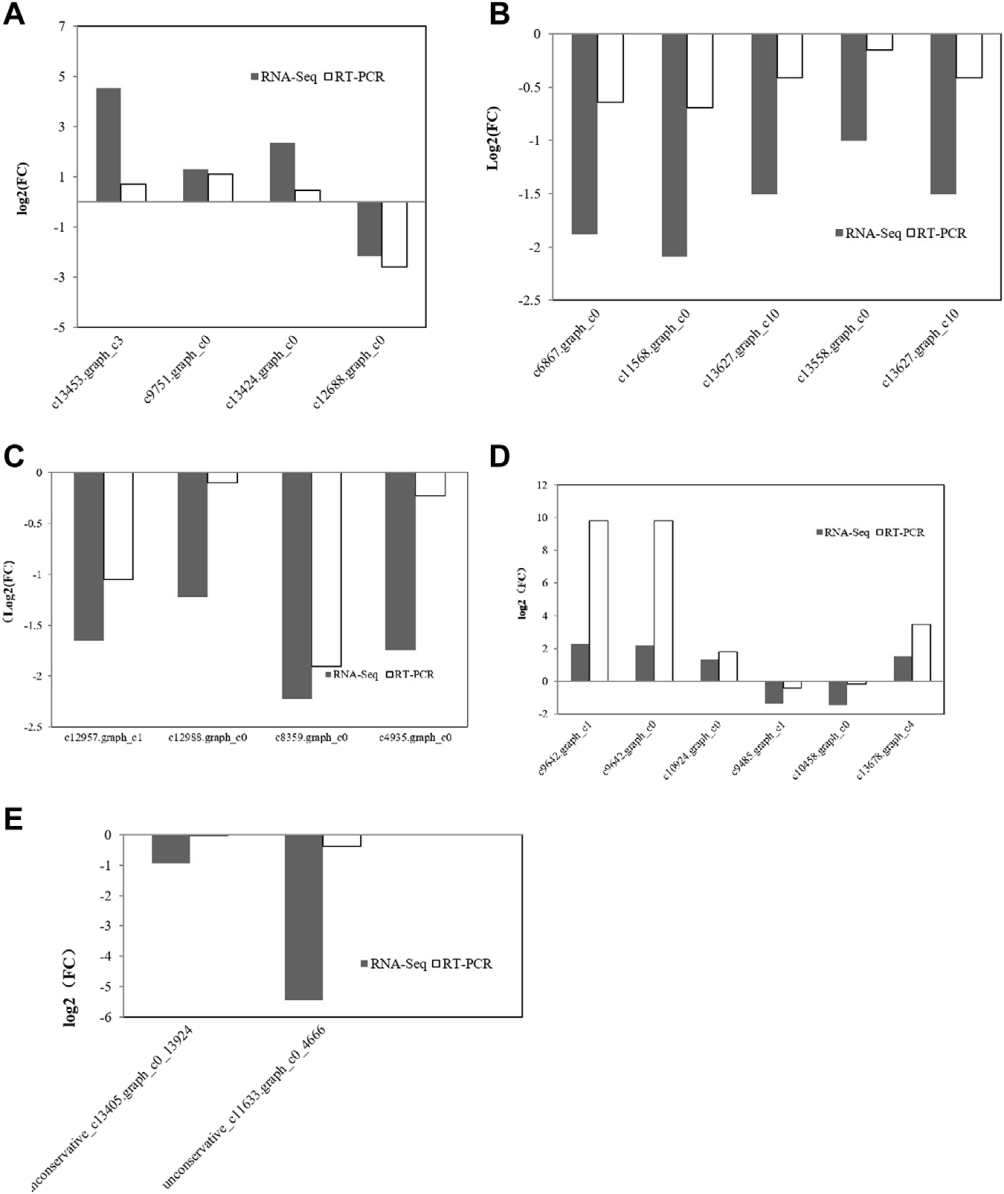
Validation of mRNA sequencing data for **(A)** nitrogen metabolism, **(B)** photosynthesis metabolism, **(C)** glycometabolism, and **(D)** amino acid metabolism via qRT-PCR. **(E)** Validation of miRNA sequencing data via qRT-PCR.

miRNAs can regulate gene expression through destabilization of the mRNA and translational repression (Chung et al., 2017). Most miRNAs are involved in regulating the biosynthesis of secondary metabolites, followed by protein metabolism (Azaman et al., 2020). From the above results, it can be seen that the transcription level of genes related to amino acid synthesis in *C. pyrenoidesa* is regulated by miRNAs, and miRNAs have a certain influence on nitrogen entering *C. pyrenoidesa* to synthesize amino acids. To validate the sequencing results, 26 mRNAs and two miRNAs were selected for qRT-PCR validation. Expressions of 20 mRNAs and two miRNAs were determined and compared with the RNA-seq data, which was similar to our sequencing data including seven upregulated and 12 downregulated mRNAs (Figure 6). Furthermore, the expression of unconservative_c13405.graph_c0_13924 and the unconservative_c11633.graph_c0_4666 was downregulated (Figure 6), which closely matched with the sequencing data. The results showed that miRNA and mRNA data were credible.

## 4 Conclusion

In this study, the peak biomass yield was reached at a 3:1 inoculation ratio of *Y. lipolytica* to *C. pyrenoidosa*. The co-culture system promoted the removal of COD, TN, and NH_3_-N, particularly at 3:1 ratio, the removal of COD reached 56.25% (675 mg/L) and the removal of both TN and NH_3_-N could reach 90.27% (147.82 mg/L and 98.54 mg/L, respectively) after 6 days of co-culture. *Y. lipolytica* had an effect on the transcriptome of *C. pyrenoidosa*, especially with the co-culture of *C. pyrenoidosa* and *Y. lipolytica* at a 3:1 ratio after 3 days. Therefore, we focused on *Y. lipolytica*-responsive mRNAs and miRNAs by performing transcriptome sequencing and RNA-seq of *C. pyrenoidosa* after 3 days. Our sequencing results revealed a large number of *Y. lipolytica*-responsive known miRNAs or novel miRNAs in *C. pyrenoidosa*, which regulate the target gene expression. The transcript levels of most of the genes related to nitrogen metabolism, carbon metabolism, amino acid synthesis, and other transporter proteins were upregulated in the co-culture system of *C. pyrenoidosa*. In addition, two differentially expressed miRNAs were negatively correlated with their target mRNA expressions. One of these miRNAs (unconservative_c13405.graph_c0_13924) regulates gene expression of cysteine dioxygenase, hypothetical protein, and histone-lysine N-methyltransferase SETD1, thereby reducing amino acid metabolic capacity; the other miRNA (unconservative_c11633.graph_c0_4666) may promote upregulation of genes encoding the ATP-binding cassette, subfamily C (CFTR/MRP), member 10 (ABCC10), thereby promoting nitrogen and carbon transport in *C. pyrenoidosa*. Based on the present results, we propose a pollutant disposal model for the mode of action of *Y. lipolytica* in *C. pyrenoidosa*. It seems that *Y. lipolytica* may function as a stress inducer to promote the removal of nitrogen and carbon via nitrogen, photosynthesis, and amino acid metabolism, as well as glycometabolism, which further prompts the activation of a signaling cascade of miRNAs. Our results may provide a valuable resource for further investigation of the regulation mechanisms of *Y. lipolytica* on the removal of COD, TN, and NH_3_-N.

## Data Availability

The data presented in the study are deposited in the GEO repository, accession number GSE231399.
