# Supplementary material for: Enhanced nitrogen removal via Yarrowia lipolytica-mediated nitrogen and related metabolism of Chlorella pyrenoidosa from wastewater
Source: Front Bioeng Biotechnol. 2023 Jun 22;11:1159297. doi: 10.3389/fbioe.2023.1159297 (PMC10325826; doi:10.3389/fbioe.2023.1159297)
Supplement: Supplementary file 1 [file Table1.DOCX]

Supplementary Material

**Enhanced nitrogen removal via *Yarrowia lipolytica*-mediated nitrogen and related metabolism of *Chlorella pyrenoidosa* from wastewater**

Yuming Zhong, Danni Lin, Sufen Li, Qin Wang, Hui Liu, Lukai Ma, Huifan Liu*

*** Correspondence:** Huifan Liu: lm_zkng@163.com

# Supplementary Tables

**Table S1. Primer sequences of reference and target genes**

| The name of primer | Sequence |
| --- | --- |
| c10458.graph_c0-1-F | CTCCTCAGCCTTCACCTTCTC |
| c10458.graph_c0-1-R | ACCGCCATCAAGCCCATC |
| c10924.graph_c0-1-F | ACGCTCACGGTCACAATG |
| c10924.graph_c0-1-R | TGCCATCTTCCACCACTTC |
| c11568.graph_c0-1-F | TTGTCGGTCTGGATGATG |
| c11568.graph_c0-1-R | AAGATTGTGAGCGTGGAG |
| c12688.graph_c0-1-F | CTGACGATGAATAATGTG |
| c12688.graph_c0-1-R | TGCTAAAGATGTTGGTTA |
| c12957.graph_c1-1-F | CACCACCACGATGCTCAG |
| c12957.graph_c1-1-R | CCTCACCCTCCTTCTCAAAC |
| c12988.graph_c0-1-F | GTCCAGCAGGTCCAGCATCA |
| c12988.graph_c0-1-R | GCCAAGCAGCAGTCGTTCATC |
| c13424.graph_c0-1-F | AATCTTGGCGTTGAACAT |
| c13424.graph_c0-1-R | TATGGTCATCGGCTTCTC |
| c13453.graph_c3-1-F | TATCCACTCAATCCACAGTATT |
| c13453.graph_c3-1-R | GTTACGCACTCTGGTTCT |
| c13558.graph_c0-1-F | ATTCTTTCTGTGGTCTGT |
| c13558.graph_c0-1-R | GTATGAACGCATCTAAGTTA |
| c13627.graph_c10-1-F | AAGAAGAAGGAGGTGGTG |
| c13627.graph_c10-1-R | ATCAGCAGGTTCAGGAAG |
| c13678.graph_c4-1-F | TTCTGCGATTCCCACTAT |
| c13678.graph_c4-1-R | CCCTTCTTCCAACAAATGA |
| c4935.graph_c0-1-F | AAGAACCCTCTCAATCCT |
| c4935.graph_c0-1-R | GTGCGAACAACAAGAATC |
| c6867.graph_c0-1-F | GACGATGGTGAATTGGTT |
| c6867.graph_c0-1-R | CGCATCAGGTATTCTTCC |
| c8359.graph_c0-1-F | CATCAACATCGGCTTCAG |
| c8359.graph_c0-1-R | GCATGAGGTAGACAACAG |
| c9485.graph_c1-1-F | ATTGATAAGCGACCTGAG |
| c9485.graph_c1-1-R | CGGAAAGGAATGAAATGA |
| c9642.graph_c0-1-F | GGGTCGTTGGTGCTCAAG |
| c9642.graph_c0-1-R | GCTCCACTGCTCCATATTCTG |
| c9751.graph_c0-1-F | AAAGGGAACATACAATAGGA |
| c9751.graph_c0-1-R | CTAAACCAACCGTCAAAG |
| unconservative_c10724.graph_c1_1848(reference gene)-RT | GTCGTATCCAGTGCAGGGTCCGAGGTATTCGCACTGGATACGACAGGAGG |
| unconservative_c10724.graph_c1_1848(reference gene)-F | TTGCGGCTGAACACCTCC |
| unconservative_c13405.graph_c0_13924-RT | GTCGTATCCAGTGCAGGGTCCGAGGTATTCGCACTGGATACGACTGCAAT |
| unconservative_c11633.graph_c0_4666-RT | GTCGTATCCAGTGCAGGGTCCGAGGTATTCGCACTGGATACGACGCCCTG |
| unconservative_c10724.graph_c1_1848-F1 | GACGTGCGGCTGAACACC |
| unconservative_c13405.graph_c0_13924-F1 | GCGGCGGAGTGACGAA |
| unconservative_c11633.graph_c0_4666-F1 | CGCCCAGGTGCTGCAG |

**Table S2. Transcriptome sequencing summary of *C. pyrenoidosa* with (CY) and without (C) *Y. lipolytica* co-culture**

| Sample | Read Number | Base Number | GC Content | ≥ Q30 |
| --- | --- | --- | --- | --- |
| CY-3day-01 | 48,987,815 | 14,636,602,692 | 65.67% | 94.37% |
| CY-3day-02 | 61,407,284 | 18,369,114,146 | 65.73% | 94.10% |
| CY-3day-03 | 47,290,789 | 14,139,989,226 | 65.76% | 94.33% |
| C-3day-01 | 52,342,976 | 15,662,388,878 | 66.30% | 94.06% |
| C-3day-02 | 55,882,497 | 16,712,074,408 | 66.26% | 94.25% |
| C-3day-03 | 45,937,405 | 13,735,314,896 | 66.35% | 94.07% |
| CY-5day-01 | 42,306,053 | 12,659,368,934 | 66.26% | 94.16% |
| CY-5day-02 | 49,351,950 | 14,764,678,772 | 66.30% | 94.03% |
| CY-5day-03 | 55,163,320 | 16,492,922,882 | 66.26% | 94.31% |
| C-5day-01 | 54,588,755 | 16,323,615,618 | 66.35% | 93.82% |
| C-5day-02 | 62,514,501 | 18,686,445,726 | 66.40% | 93.84% |
| C-5day-03 | 47,836,029 | 14,295,990,566 | 66.46% | 94.08% |

**Table S3. Assembled data for transcripts and unigenes in the transcriptome**

| Length range | Transcripts | Unigenes |
| --- | --- | --- |
| 200–300 | 12,670 (4.91%) | 10,885 (32.01%) |
| 300–500 | 11,548 (4.47%) | 6,648 (19.55%) |
| 500–1000 | 24,851 (9.63%) | 6,433 (18.92%) |
| 1000–2000 | 56,274 (21.80%) | 5,912 (17.38%) |
| 2000+ | 152,804 (59.19%) | 4,132 (12.15%) |
| Total Number | 258,147 | 34,010 |
| Total Length | 709,937,024 | 31,783,370 |
| N50 Length | 3,792 | 1,704 |
| Mean Length | 2750.13 | 934.53 |

**Table S4. Functional annotation in the database**

| DEG Set | Annotated | COG | GO | KEGG | KOG | Pfam | Swiss-Prot | eggNOG | nr |
| --- | --- | --- | --- | --- | --- | --- | --- | --- | --- |
| Z3d01_Z3d02_Z3d03_vs_G3d01_G3d02_G3d03 | 8298 | 3115 | 5925 | 3182 | 4566 | 5752 | 3512 | 6647 | 8181 |
| Z5d01_Z5d02_Z5d03_vs_G5d01_G5d02 | 3203 | 1451 | 2246 | 1405 | 1884 | 2452 | 1622 | 2626 | 3155 |
